# HIP1R acts as a tumor suppressor in gastric cancer by promoting cancer cell apoptosis and inhibiting migration and invasion through modulating Akt

**DOI:** 10.1002/jcla.23425

**Published:** 2020-06-16

**Authors:** Jinliang Zhu, Xin Wang, Huiyuan Guan, Qiong Xiao, Zhonghua Wu, Jinxin Shi, Fei Zhang, Peng Gao, Yongxi Song, Zhenning Wang

**Affiliations:** ^1^ Key Laboratory of Precision Diagnosis and Treatment of Gastrointestinal Tumors Department of Surgical Oncology and General Surgery Ministry of Education The First Affiliated Hospital of China Medical University Shenyang China; ^2^ Department of Gastrointestinal Surgery Shenyang Anorectal Hospital Shenyang China

**Keywords:** apoptosis, gastric cancer, huntingtin**‐**interacting protein 1**‐**related, invasion, migration

## Abstract

**Background:**

Huntingtin‐interacting protein 1‐related (HIP1R) is a multi‐domain gene that exerts many cellular functions including altering T cell–mediated cytotoxicity and controlling intracellular trafficking. However, its clinical significance and function in gastric cancer (GC) have not been described.

**Methods:**

The expression levels of HIP1R were tested by the transcriptional and translational expression analysis and immunohistochemistry (IHC) in matched adjacent non‐tumorous vs tumor tissue specimens. The biological function of HIP1R on apoptosis, migration, and proliferation was evaluated by flow cytometry, Transwell, Cell Counting Kit‐8 (CCK‐8) assays, colony formation assays, and EdU labeling assays, respectively.

**Results:**

We found downregulated HIP1R in GC compared with adjacent non‐tumorous tissue, and HIP1R expression associated with N classification. We further found that the expression of HIP1R could induce apoptosis and inhibit proliferation, migration, invasion of GC cells, possibly through modulating Akt.

**Conclusions:**

Our data indicate that HIP1R may act as a potential diagnostic biomarker and a tumor suppressor gene in GC, potentially representing a novel therapeutic target for future GC treatment.

## 1 INTRODUCTION

Gastric cancer (GC) remains a highly widespread malignancy worldwide, with 783 000 patient deaths from GC in 2018 ranking it 3rd among cancer**‐**related deaths.^1^ Clinical symptoms of GC often appear late in the disease development, most patients are diagnosed at an advanced stage, resulting in limited treatment choices. Furthermore, its high postoperative recurrence and distant metastasis rates are leading causes for poor prognosis. Thus, identifying new biomarkers and potential targets for GC treatment remains urgent needs.^2^, ^3^

Huntingtin**‐**interacting protein 1**‐**related (HIP1R)^4^, ^5^ belongs to an evolutionarily conserved family^6^ along with yeast Sla2p^7^, ^8^ and mammalian HIP1 proteins.^9^ HIP1 and HIP1R are reported to be components of the clathrin**‐**mediated endocytosis pathway and have known functions in endocytosis and actin cytoskeleton regulation.^10^, ^11^, ^12^, ^13^, ^14^, ^15^ HIP1R is the only known mammalian relative of HIP1, and previous reports indicate that HIP1 and HIP1R expression alterations may contribute to cell growth and survival.^16^ HIP1 has been reported to affect tumorigenesis in many tumor types;^17^, ^18^, ^19^, ^20^, ^21^ however, HIP1R has rarely been reported on in tumors. Previous studies suggest that HIP1R may be a key factor in maintaining chromosome integrity. In HIP1R**‐**deficient cells, chromosomes mismatched and eventually produced multinucleated cells, indicating that HIP1R is essential for tumorigenesis or other human diseases.^22^ Additionally, recent studies found that HIP1R plays a significant role in tumor immunotherapy by modulating T cell**–**mediated cytotoxicity via lysosomal degradation of PD**‐**L1.^23^ Low expression of HIP1R is associated with worse prognosis in diffuse large B**‐**cell lymphoma (DLBCL) patients with the treatment of rituximab**‐**CHOP regimens.^24^, ^25^ Ectopic HIP1R expression has also been demonstrated to promote migration and invasion of less**‐**invasive prostate cancer cells.^26^ Although HIP1R associates with human cancer biology, its precise role in GC progression remains unclear. Therefore, our study was designed to explore the clinicopathological associations, biological functions, and possible regulatory mechanisms of HIP1R in GC.

## 2 MATERIALS AND METHODS

### 2.1 Patients and samples

All patient specimens were gathered with signed informed consent from the patients, and ethics approval for the study was gained from the ethics committee of the research institute. A total of 580 GC tissue samples were collected, including 380 paired adjacent non**‐**tumorous vs tumor samples from patients who experienced curative radical gastrectomy with lymph node excision at the First Affiliated Hospital of China Medical University between 2009 and 2014. Postoperative pathological reports were obtained including age, gender, the extent (size) of the tumor, differentiation, lymphatic invasion, venous invasion, and TNM stage. The TNM classification system criteria for GC were classified according to the 8th edition of the American Joint Committee on Cancer (AJCC) staging manual. All patients’ clinicopathological data are presented in Table 1.

**TABLE 1:** Correlation between HIP1R expression and clinicopathologic characteristics

Characteristics	Number	HIP1R low expression (%)	HIP1R high expression (%)	*P*‐value
Total	580	267	313	
Age (y)^a^				
≤60	289	132 (45.7)	157 (54.3)	.862
>60	291	135 (46.4)	156 (53.6)	
Gender				
Male	421	190 (45.1)	231 (54.9)	.477
Female	159	77 (48.4)	82 (51.6)	
Pathophysiologic features				
Tumor size (cm)^b^				
≤5	290	139 (47.9)	151 (52.1)	.359
>5	290	128 (44.1)	162 (55.9)	
Histological grade				
Well	47	15 (31.9)	32 (68.1)	.060
Moderate	116	49 (42.2)	67 (57.8)	
Poor	417	203 (48.7)	214 (51.3)	
T classification				
T1 + T2	40	18 (45.0)	22 (55.0)	.892
T3 + T4	540	249 (46.1)	291 (53.9)	
N classification				
N0	130	51 (39.2)	79 (60.8)	**.012**
N1	122	47 (38.5)	75 (61.5)	
N2	122	57 (46.7)	65 (53.3)	
N3	206	112 (54.4)	94 (45.6)	
Metastasis				
M0	557	257 (46.1)	300 (53.9)	.802
M1	23	10 (43.5)	13 (56.5)	
TNM stage				
I	23	12 (52.2)	11 (47.8)	.062
II	133	48 (36.1)	85 (63.9)	
III	392	193 (49.2)	199 (50.8)	
IV	32	14 (43.8)	18 (56.2)	
Lymphatic invasion				
Negative	387	170 (43.9)	217 (56.1)	.149
Positive	193	97 (50.3)	96 (49.7)	
Venous invasion				
Negative	575	264 (45.9)	311 (54.1)	.529
Positive	5	3 (60.0)	2 (40.0)	

^a^ The median age at surgery for this cohort was 60 y.

^b^ The median size of the primary tumor for this cohort was 5 cm.

Bold values are indicates statistical significance (*p*<.05).

### 2.2 Cell cultivation and transfection

The cells used in this study included AGS, HGC27, SGC7901, MGC803, and one normal gastric epithelium cell line (GES1). All cells were obtained from the cell line library of the Chinese Academy of Sciences. The cell culture conditions were as follows: the medium was RPMI 1640 medium (Invitrogen) with 10% fetal bovine serum, the temperature in the wet incubator was 37°C, and it contained 5% carbon dioxide (Thermo).

For HIP1R overexpression, the CMV‐MCS‐IRES‐EGFP‐SV40‐Neomycin plasmid was obtained from Genechem. Empty vector was used as a negative control. The small interfering RNA (siRNA) against HIP1R and control siRNA were obtained from Syngen Tech. GC cells were transfected with plasmid or with HIP1R siRNA using lipofectamine 3000 Reagent (Invitrogen) following the instructions on the manual. Cells were collected 48 hours after transfection. Quantitative real‐time PCR (qRT‐PCR) or Western blotting was used to monitor transfection efficiency.

### 2.3 qRT‐PCR

Total RNA was isolated from cultured cells using TRIzol reagent (Invitrogen). RNA was reverse**‐**transcribed into cDNA using the Reverse Transcription Kit (TaKaRa, RR047A) as instructed by the manufacturer. qRT**‐**PCR was carried out in a LightCycler 480 II Real**‐**Time PCR system (Roche) using TB Green (TaKaRa, RR820A). GAPDH served as an internal mRNA control. The comparative Ct method was applied to determine relative RNA expression. Primer sequences are listed below (forward and reverse, 5′**‐**3′).

HIP1R, CCTTCTGGTCCTATGCCATTG, AATGTCCCCACAGGTCTCCAA; GAPDH, CGGATTTGGTCGTATTGGG, CTGGAAGATGGTGATGGGATT.

### 2.4 Western blotting

Total proteins from transfected GC cells were extracted via the Total Protein Extraction Kit (KeyGEN). The BCA protein assay was performed for protein quantification from the total extracted cell protein lysates, then the same**‐**volume of protein samples were added into the SDS**‐**PAGE and transferred onto PVDF membranes (Millipore). The proteins on the membrane were then blocked with 5% skim milk for 2 hours and incubated overnight at 4°C with anti**‐**HIP1R (BD#612118, 1:1000), anti**‐**Phospho**‐**Akt (S473) (CST#4060, 1:1000), anti**‐**Akt (CST#9272, 1:1000), anti‐mTOR (CST#2983, 1:1000), anti‐p‐mTOR (S2448) (CST#5536, 1:1000), anti‐Bak (SAB#48813, 1:1000), anti‐Cleaved‐Caspase‐9 (SAB#40504, 1:1000), anti‐E‐cadherin (SAB#48355, 1:1000), anti‐Vimentin (SAB#41532, 1:500), and anti‐β‐Actin (SAB#21800, 1:3000) antibodies. The membranes were then incubated with the corresponding secondary antibody (ZSGB‐BIO, ZB‐2305 or ZB‐2301, 1:5000) at room temperature for 1.5 hours. The proteins were visualized using GelCapture software (DNR Bio**‐**Imaging Systems) with β‐Actin as the internal reference.

### 2.5 Tissue microarrays (TMAs) and IHC

The representative areas of each formalin**‐**fixed paraffin**‐**embedded tissue cylinder (typical diameter: 2 mm) were punched from different primary GC blocks (cores of intratumoral or peritumoral tissues from “donor” blocks) and collected into one empty “recipient” wax block like a microarray (4 cm × 2 cm) as previously described.^27^, ^28^ TMA tissue blocks were sliced into 4**‐**μm thick sections. Following conventional dewaxing in xylene, rehydration through graded alcohols, and blocking of endogenous peroxidase in 3% peroxyl for 10 minutes, sections were heated in Tris**‐**EDTA buffer (PH: 9.0) for antigen recovery. After blocking with goat serum (SP**‐**9002, ZSGB**‐**BIO), sections were incubated with antibodies against HIP1R (BD#612118, diluted 1:400) and p‐Akt (CST#4060, diluted 1:400) overnight at 4°C. Goat anti**‐**mouse or anti**‐**rabbit IgG (SP**‐**9002/9001, ZSGB**‐**BIO) was used for incubation with sections for 15 minutes at room temperature. Finally, sections were visualized using DAB (ZLI**‐**9010, ZSGB**‐**BIO) and hematoxylin.

A digital section scanner (KF**‐**PRO**‐**005) was used to scan all sections to generate digital images. The images could be viewed at 400× magnification with high fidelity. Two independent blinded researchers randomly analyzed each digital sample image. Following a previous semi**‐**quantitative scoring method, the immunoreactivity score (IS) equaled the staining intensity score multiplied by the positive cell rate score.^29^ The staining intensity score was defined as 0, negative; 1, weak; 2, moderate; 3, strong. The percentage of stained cells was defined as: 0, <5%; 1, 5%**‐**25%; 2, 26%**‐**50%; 3, 51%**‐**75%; and 4, >75%. The IS ranges from 0 to 12. A score of 0 as negative, 1**‐**3 as weakly positive, 4**‐**8 as moderately positive, 9**‐**12 as strongly positive. Tissues were then separated into high HIP1R expression (IS ≥ 4) and low expression (IS < 4) groups. This IHC scoring system has been used in our previous studies.^30^, ^31^

### 2.6 Cell proliferation assays

#### 2.6.1 CCK8 assays

Forty‐eight hours after transfection with HIP1R or negative control, approximately 2 × 10^3^/well cells were inoculated in 96**‐**well culture plates. After cells adhered, ten μl of CCK**‐**8 solution (Kumamoto) was added to each well for 1 hour at 37°C. The optical density of cells was then detected using a microplate reader (Bio**‐**Rad) at a wavelength of 450 nm. The same measurement was repeated every 24 hours until the last plate was detected.

#### 2.6.2 Colony formation assays

Cells were plated at 6‐well plates (1000 cells/well) and incubated for 1 week. The cells were fixed with methanol for 10 minutes and then stained with 1% crystal violet. Colonies were manually counted for more than 50 cells using ImageJ. The experiment was carried out at least three times.

#### 2.6.3 EdU labeling assays

Cell proliferation was determined using the BeyoClick™ EdU Kit (Beyotime Biotechnology) following the manufacturer's instructions. Briefly, cells were plated at 12‐well plates (1 × 10^5^ cells/well) and incubated with equal volumes of the EdU work solution for 2 hours at 37°C. Then, cells were fixed with 4% paraformaldehyde for 15 minutes. The stained cells were examined with Leica LMD6500 fluorescence microscope and randomly photographed with 10 fields.

### 2.7 Transwell assays

Cell migration and invasion abilities were measured using HGC27 and MGC803 cells 48 hours after transfection. Cells (5 × 10^4^) were added into the upper chambers for migration assays or inoculated into a Matrigel**‐**coated (BD Biosciences) invasion chamber for invasion assays. Cells were cultured in serum**‐**free media with the lower chambers containing 10% FBS growth media as a chemoattractant. After 48 hours, migrated and invasive cells attaching on the underside of the membrane were fixed and stained with hematoxylin and eosin. Ten fields were randomly imaged from the underside of the membrane using a Leica DM4000B microscope and the number of cells was counted with ImageJ.

### 2.8 Flow cytometry

Apoptosis was measured using the manufacturer's method (KeyGEN). Briefly, cells were collected 48 hours after transfection then washed with PBS. Annexin V APC and propidium iodide (PI) staining were used to assess the apoptosis rate of cancer cells within 30 minutes after staining. Each sample was then analyzed by BD FACSCalibur flow cytometry system (BD) using Cell**‐**Quest software.

### 2.9 Bioinformatics analysis

RNASeqV2 profile datasets for 375 patients with GC were downloaded from The Cancer Genome Atlas (TCGA). The dataset was split into two groups based on the HIP1R expression level. Gene Set Enrichment Analysis (GSEA) was applied using the clusterProfiler R package.^32^

### 2.10 Statistical analysis

SPSS Standard version 25.0 and GraphPad Prism 7.0 were used for statistical analysis. The appropriate non**‐**parametric *χ* ^2^ test was performed to assess HIP1R expression and clinicopathological factors. Student's *t* test or Kruskal**‐**Wallis test was used to analyze statistical significance. *P* < .05 was set as statistically significant.

## 3 RESULTS

### 3.1 HIP1R is downregulated in GC

**FIGURE 1:**
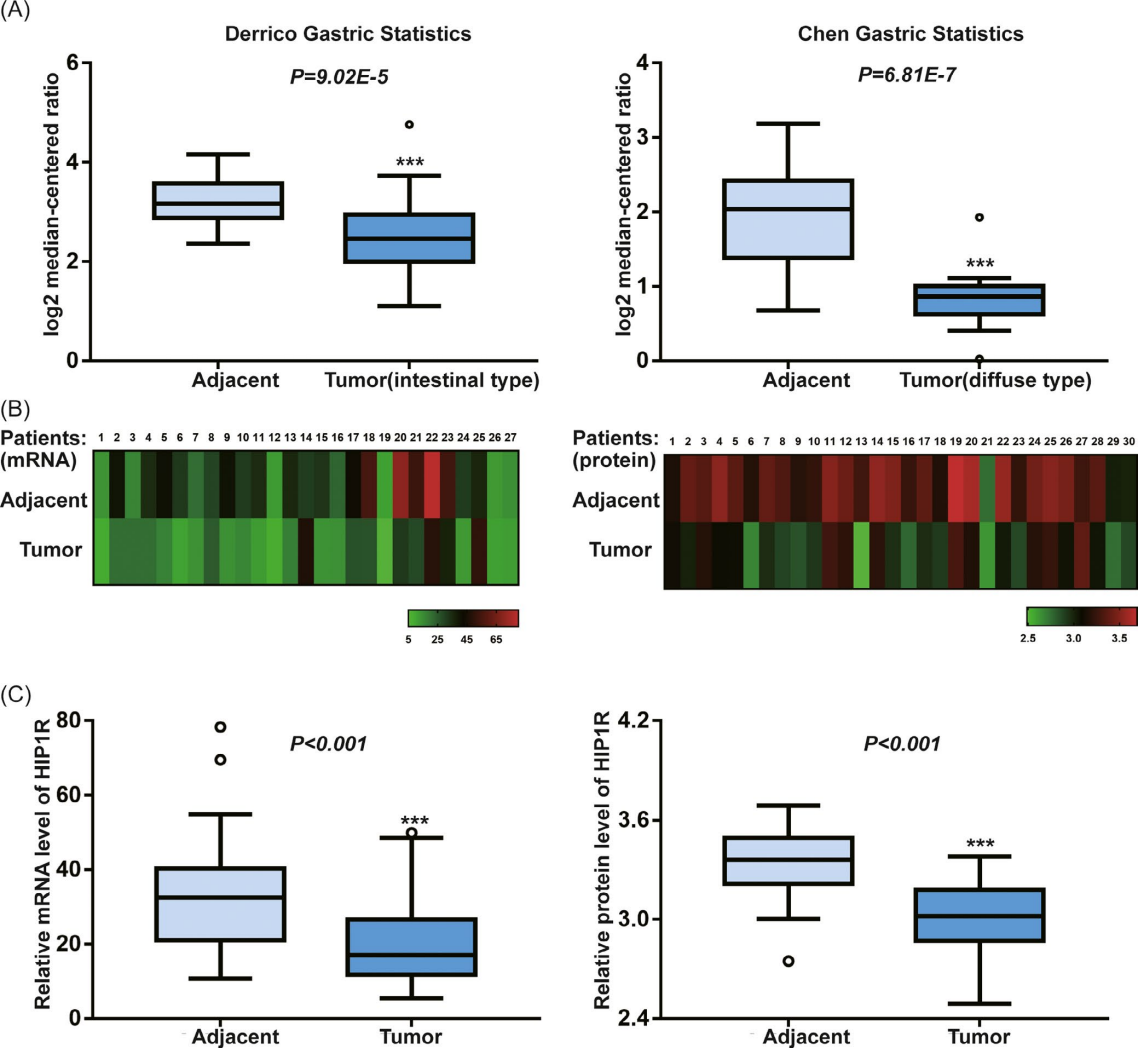
A, HIP1R mRNA is significantly downregulated in human gastric cancer compared with adjacent non‐tumor tissue in multiple datasets. Gastric cancer mRNA analyses from Oncomine were surveyed for HIP1R in gastric cancer as compared with adjacent non‐tumor tissue. HIP1R levels graphed as log2 median‐centered ratio are listed for two separate significant studies: Chen Gastric Statistics, 24 adjacent non‐tumor samples, 13 diffuse‐type tumor samples. Derrico Gastric Statistics, 31 adjacent non‐tumor samples, 26 intestinal‐type tumor samples. B, C, The expression of HIP1R on mRNA and protein levels are significantly downregulated in 30 matched human gastric cancer compared with adjacent non‐tumor tissue by transcriptional and translational expression analysis (for mRNA level three samples were not available) (*P < .05, **P < .01, ***P < .001)

Since little was published about HIP1R in GC, we decided to analyze open datasets from the Oncomine database and found downregulated HIP1R mRNA expression in human GC samples compared to normal gastric samples (Figure 1A). To further explore HIP1R expression in GC, we conducted transcriptional and translational expression analyses on GC samples and matched adjacent non**‐**tumorous samples. The results revealed that HIP1R mRNA and protein levels were downregulated in primary GC tissues (mRNA: adjacent 34.12 ± 17.013 vs tumor 20.99 ± 12.371, *P* < .001; Protein: adjacent 3.33 ± 0.200 vs tumor 2.99 ± 0.211, *P* < .001) (Figure 1B,C).

**FIGURE 2:**
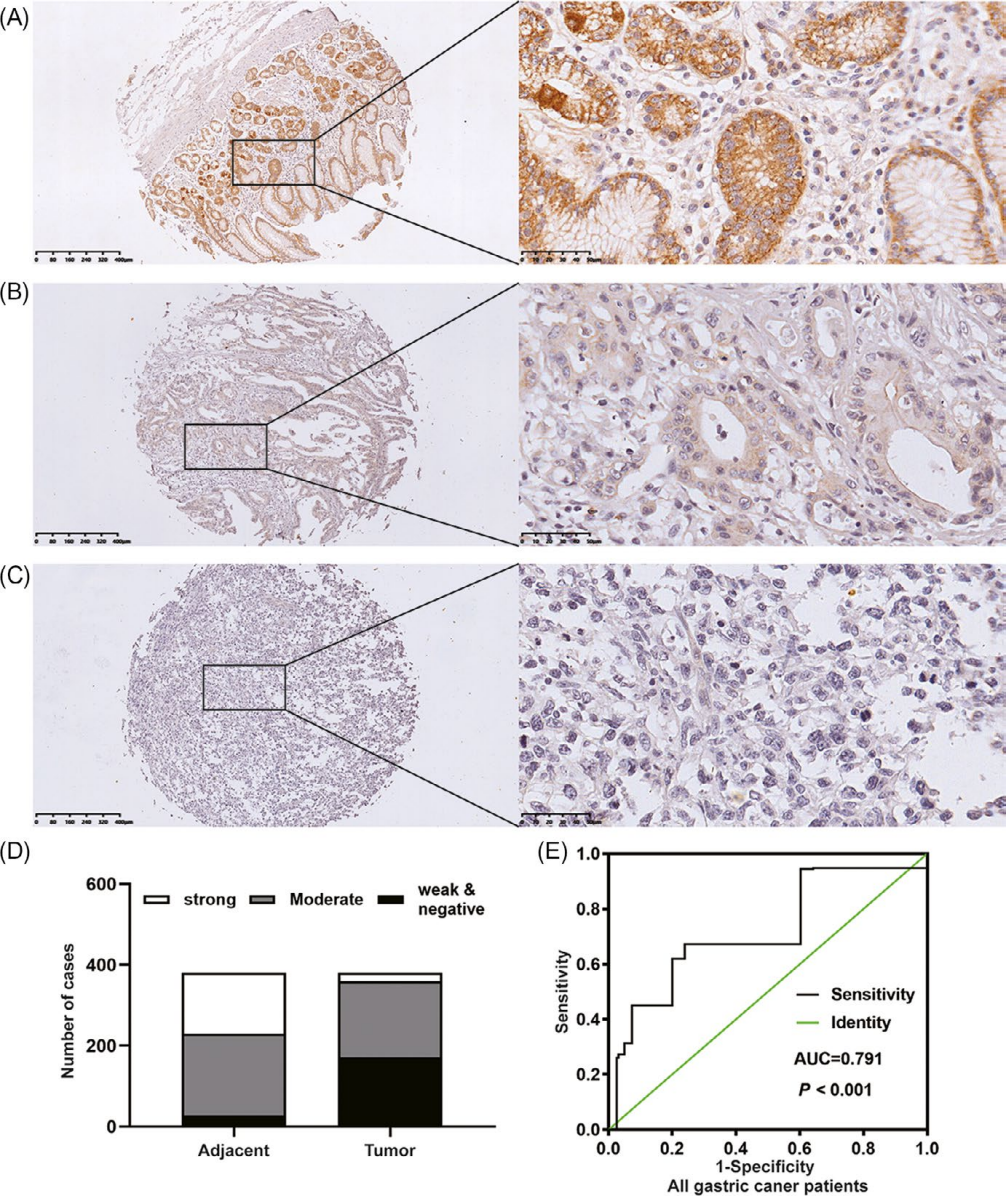
The expression of HIP1R protein was examined by IHC staining in non‐tumor gastric tissue, intestinal‐type gastric cancer and diffuse‐type gastric cancer. Representative IHC images of HIP1R are shown. A, Adjacent non‐tumor gastric mucosa, moderate to strong staining. B, C, Weak to no staining in intestinal and diffuse‐type tumor for HIP1R in the cytoplasm (Original magnification: 100× and 400×). D, HIP1R expression in paired microarray of adjacent normal stomach mucosa and tumor tissue. E, The receiver operating characteristic (ROC) curve showed the immunoreactivity score (IS) of HIP1R to distinguish gastric cancer from healthy controls

To better understand the HIP1R protein expression pattern in GC and determine its clinical significance, we examined HIP1R expression in TMAs using IHC. HIP1R was expressed in the cytoplasm. In 380 matched adjacent non**‐**tumorous tissues and corresponding tumor tissues, 352 of 380 (93%) adjacent non**‐**tumorous gastric mucosa samples showed HIP1R high expression. However, high HIP1R staining was only detected in 209 of 380 (55%) cancer tissues (Figure 2A‐D).

We next analyzed the association between HIP1R expression and clinical**‐**pathological features in 580 patients with GC. As shown in Table 1, HIP1R expression level significantly correlated with N classification (*P* = .012). However, HIP1R did not correlate with the age (*P* = .862), gender (*P* = .477) or other pathological features. No significant correlations were found in Kaplan**‐**Meier survival analysis.

### 3.2 Diagnostic value of HIP1R for GC patients

**FIGURE 3:**
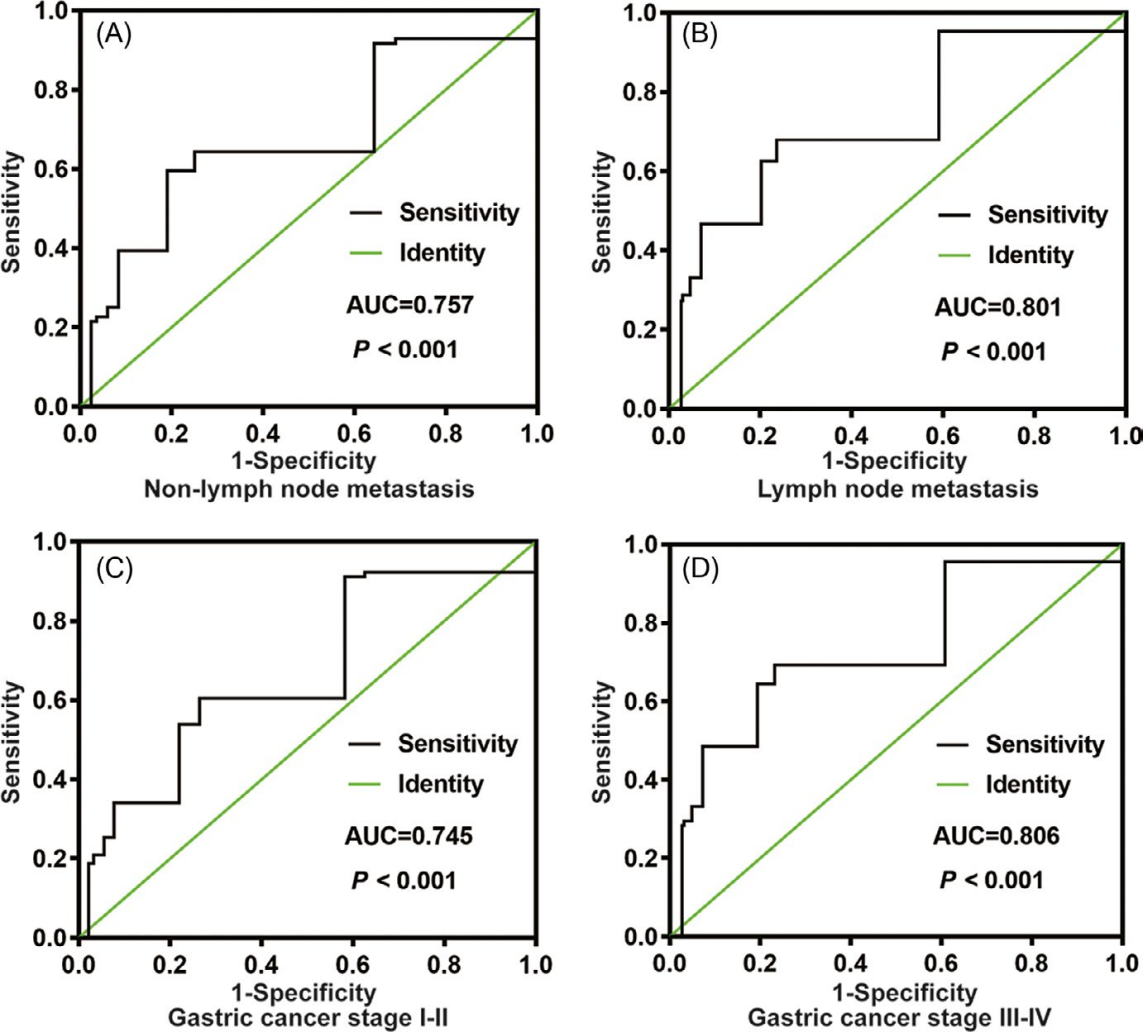
The ROC curve shows the analysis of HIP1R in the subgroup of gastric cancer patients. A, Non‐lymph node metastasis and B, Lymph node metastasis in gastric cancer from healthy controls. C, TNM stage I‐II and D, TNM stage III‐IV in gastric cancer from healthy controls

To compare the diagnostic capacity of HIP1R between controls and GC, we performed a corresponding receiver operating characteristic (ROC) curve. As shown in Figure 2E, the area under the curve (AUC) was 0.791. We then analyzed lymph node metastasis, non**‐**lymph node metastasis and TNM stage I**‐**II and III**‐**IV subgroups, finding corresponding AUC values of 0.757, 0.801, 0.745, and 0.806 respectively (Figure 3A‐D). These results indicate that HIP1R might be an appropriate diagnostic marker for GC.

### 3.3 HIP1R promotes GC cell apoptosis

**FIGURE 4:**
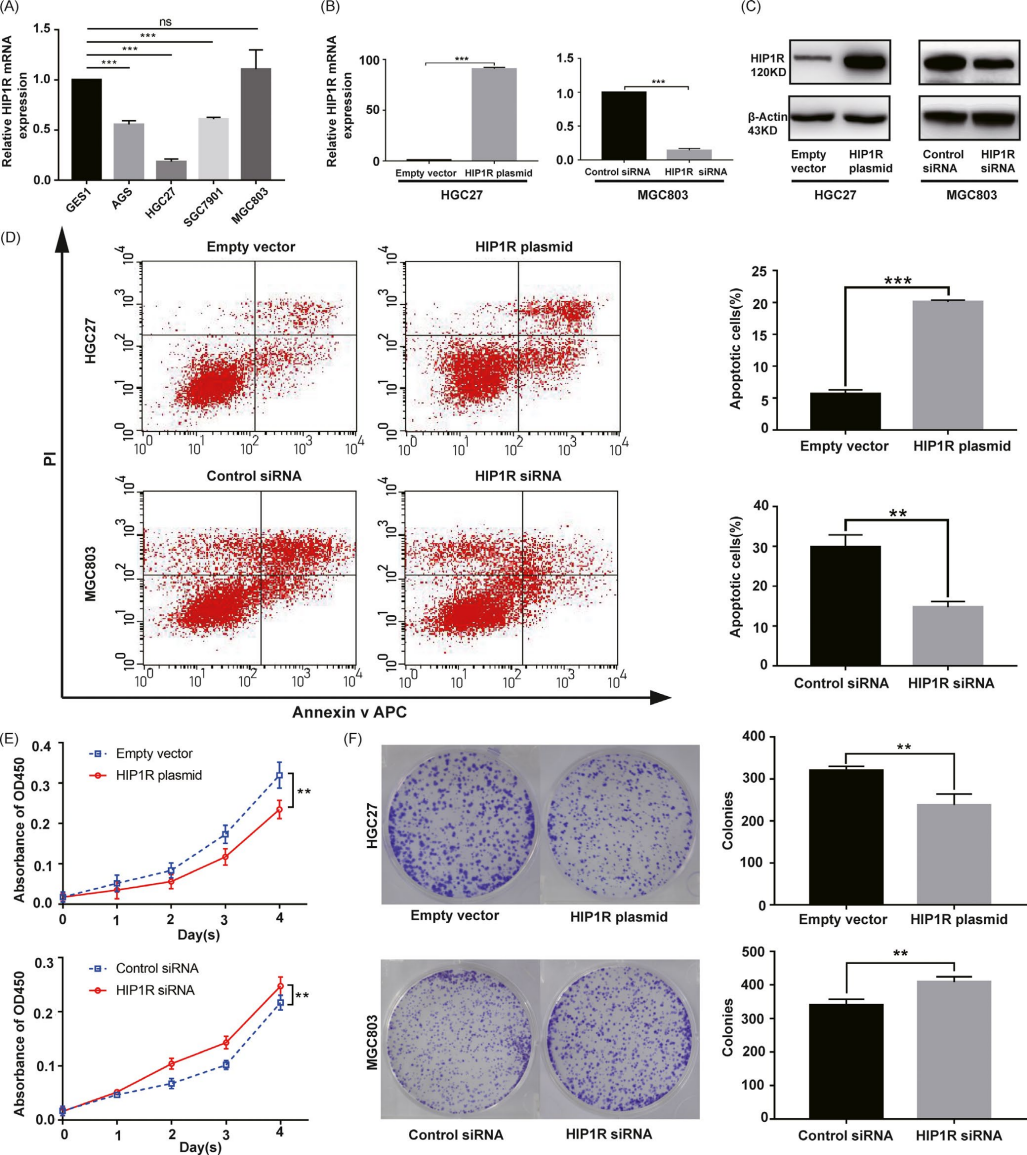
A, HIP1R mRNA levels in gastric cancer cell lines were analyzed by qRT‐PCR. B, C, Real‐time PCR and Western blot analysis showed that the overexpression and knocking down efficiencies of HIP1R in HGC27 and MGC803 cells. D, Overexpression of HIP1R promoted the apoptosis of HGC27 cells. Knocking down of HIP1R inhibited the apoptosis of MGC803 cells. Apoptotic cells were stained with Annexin V APC and PI. Histogram showed the percent of apoptotic cells in HGC27 and MGC803 groups. E, CCK8 assays revealed that overexpression of HIP1R significantly reduced the growth rate and knocking down of HIP1R increased the growth rate. F, Colony formation assays showed that HIP1R overexpression significantly reduced the mean colony number of HGC27 cells and knocking down of HIP1R increased the mean colony number of MGC803 cells. Data are shown as mean ± SD of three independent experiments

To explore the function of HIP1R in GC, we performed qRT**‐**PCR to quantify HIP1R expression in GC cell lines (Figure 4A). Compared with the GES1, HIP1R had high expression in MGC803 and had relatively low expression in three GC cell lines, especially in HGC27 (*P* < .001). Based on these results, we selected HGC27 and MGC803 for further analysis. We transfected a plasmid containing the HIP1R gene into HGC27 cells and siRNA against HIP1R gene into MGC803 cells. Next, we further validated successful transfection with qRT**‐**PCR and Western blotting (Figure 4B,C). We then tested whether HIP1R could induce apoptosis in GC cells by flow analysis for Annexin V APC and PI staining. In Figure 4D, we found that the percentage of apoptotic cells in the HIP1R plasmid group was higher than in the empty vector group (Empty vector: 5.64 ± 0.66 vs HIP1R plasmid: 20.12 ± 0.26, *P* < .001), while in the HIP1R siRNA group the percentage of apoptotic cells was lower than the control siRNA group (Control siRNA: 29.88 ± 3.00 vs HIP1R siRNA: 14.74 ± 1.42, *P* = .001).

### 3.4 HIP1R inhibits GC cell proliferation

**FIGURE 5:**
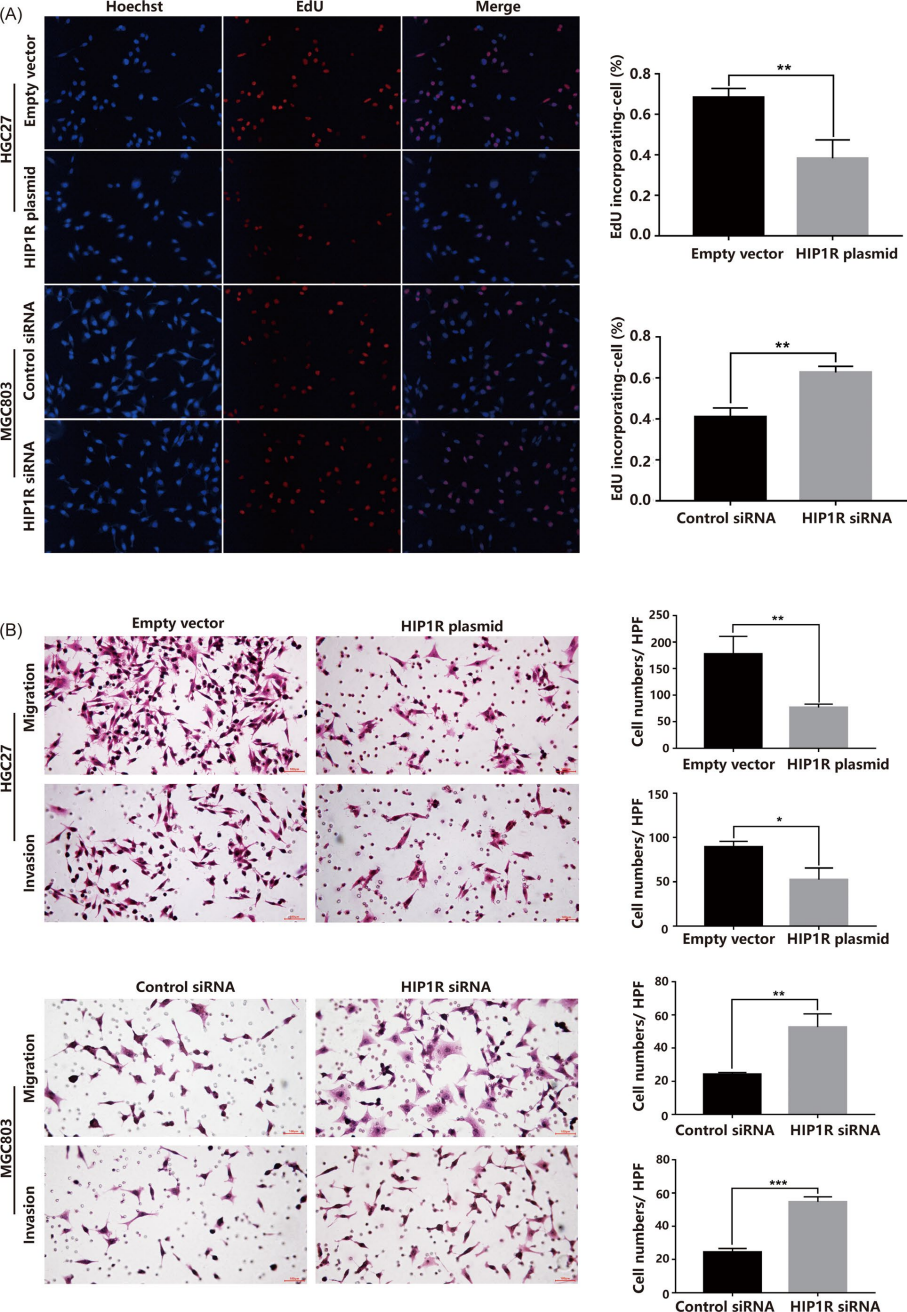
A, EdU labeling assays showed that HIP1R overexpression significantly reduced proliferation rate of HGC27 cells and knocking down of HIP1R increased the proliferation rate of MGC803 cells. B, HIP1R inhibits the capacity of migration and invasion of GC cells. Representative images are shown for Migration and Matrigel Invasion assays. Data are shown as mean ± SD of three independent experiments

To investigate the proliferation function of HIP1R in GC, we performed the Cell counting kit‐8 (CCK‐8) assays, colony formation assays and EdU labeling assays. The results were consistent and all showed that HIP1R overexpression inhibited proliferation rate of HGC27 cells, while HIP1R siRNA caused increased proliferation rate of MGC803 cells (Figures 4E,F and 5A).

### 3.5 HIP1R inhibits GC cell motility

To examine the effect of HIP1R on GC cell migration and invasion ability, we performed Transwell assay analysis. We found that HIP1R overexpression restricted GC cell migration and invasion ability compared with cells transfected with negative control. Transwell assays further showed an increased invasion ability in GC cells transfected with HIP1R siRNA compared with control siRNA group (Figure 5B).

### 3.6 The PI3K/Akt signaling pathway may be regulated by HIP1R

**FIGURE 6:**
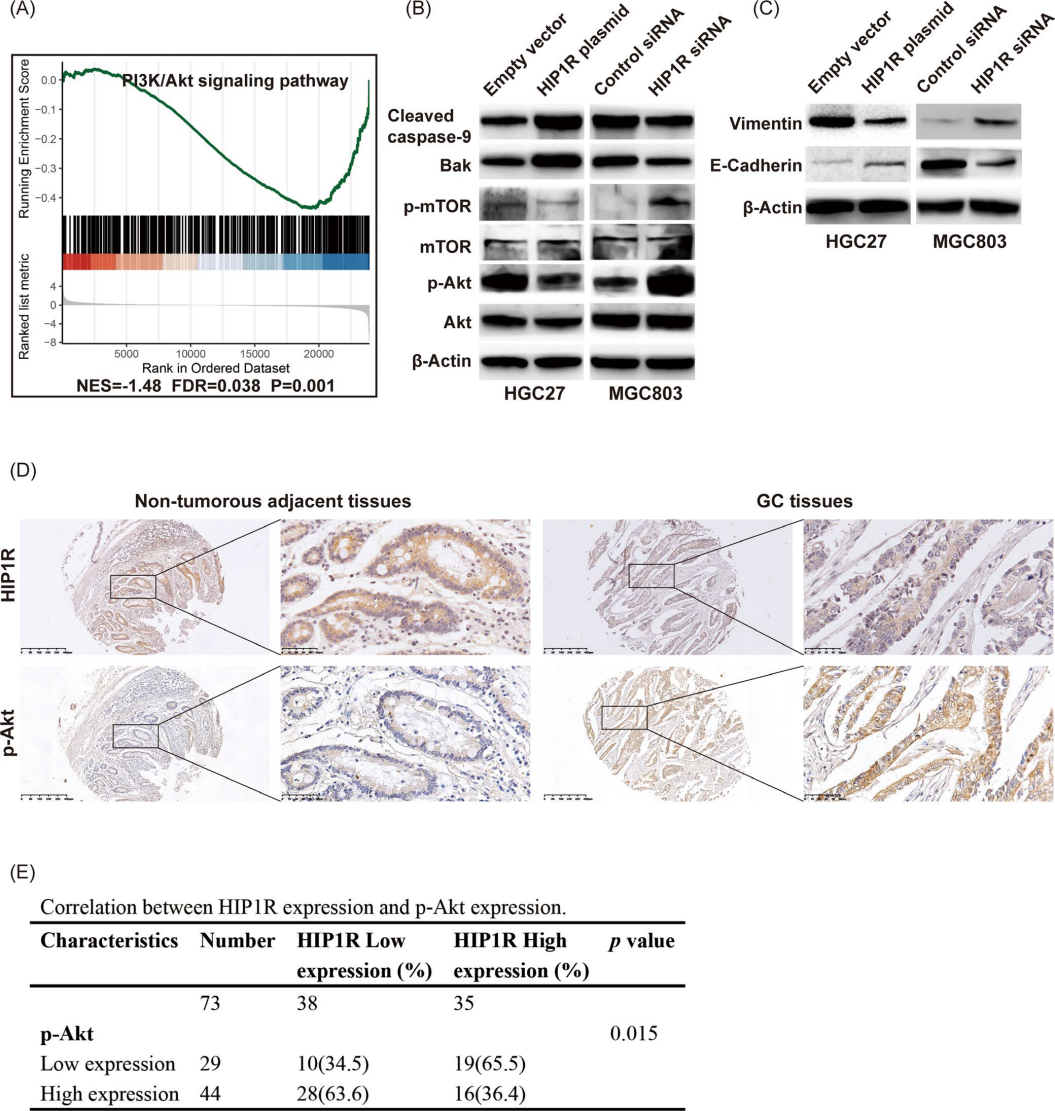
HIP1R inactivates AKT to promote apoptosis and suppress cell proliferation, migration and invasion. A, PI3K‐Akt signaling pathway was found to be correlated with HIP1R expression via gene set enrichment analysis (GSEA) on The Cancer Genome Atlas (TCGA) profiles. B, Western blotting analysis of proteins involved in PI3K/Akt signaling pathway. C, Western blotting analysis of proteins involved in EMT regulators of E‐cadherin and Vimentin. D, E, The relationship between HIP1R and p‐Akt were performed by IHC in TMA. Representative IHC images of HIP1R and p‐Akt were shown that the expression of HIP1R inversed correlated with the expression of p‐Akt

To investigate pathways participating in HIP1R**‐**mediated GC progression, GSEA was performed using published GC data from the TCGA GC database (n = 375). We found that the PI3K/Akt signal transduction pathway most correlated with HIP1R expression (Figure 6A). To verify this analysis result, we examined changes in Akt, phospho‐Akt (p**‐**Akt), mTOR, phospho‐mTOR (p‐mTOR), Cleaved Caspase‐9 and Bak by Western blotting. We found that compared with the empty vector group, the expression levels of p‐Akt, p‐mTOR were suppressed in the HIP1R plasmid group, but the expression levels of Cleaved Caspase‐9, Bak were increased. Opposing results were observed in the HIP1R siRNA group compared with the control siRNA group (Figure 6B). To investigate whether HIP1R suppresses epithelial‐mesenchymal transition (EMT) via the AKT pathway, we also examined changes of E‐Cadherin and Vimentin. We found that overexpression of HIP1R enhanced the expression of E‐cadherin and reduced the expression of Vimentin, and vice versa (Figure 6C). To further ascertain the relationship between HIP1R and p‐Akt, we performed the IHC in TMA in 73 paired samples. We found that the expression of HIP1R inversed correlated with the expression of p‐Akt (Figure 6D,E).

## 4 DISCUSSION

Gastric cancer is a commonly diagnosed malignancy and accounting for considerable global cancer morbidity and mortality. Although many abnormally expressed genes are known to contribute to malignant GC phenotypes, new therapeutic biomarkers for individual GC treatment are still urgently needed. In recent years, an increasing number of studies have indicated that derailed endocytosis is functionally relevant in tumorigenesis.^33^, ^34^, ^35^ Based on structural and functional data, HIP1R was found to participate in clathrin**‐**mediated endocytosis.^16^ So far, its expression pattern and clinical significance in GC have been ambiguous. Here, we found that HIP1R was frequently downregulated in GC specimens compared with normal specimens using transcriptional and translational expression analysis and immunohistochemistry. Our results were consistent with the available public database from Oncomine. Clinicopathological analysis showed that HIP1R was associated with N stage. Wong et al^24^, ^25^ reported that HIP1R was highly expressed in normal peripheral blood and lymphoid tissues and that lower HIP1R protein and mRNA expression significantly correlated with worse survival in DLBCL patients. These results suggest that HIP1R downregulation correlates with malignant behavior. Previous studies have also suggested that HIP1R plays important roles in gastric physiology, mucosal structure, and parietal cell secretory membrane dynamics. In Hip1r**‐**knockout mice, parietal cells were lost in the stomach mucosa which induced the evolution of spasmolytic polypeptide expressing metaplasia (SPEM).^36^, ^37^, ^38^ SPEM has been identified as precancerous metaplasia associated with GC.^39^ Thus, we speculate that HIP1R may act as a tumor suppressor for GC development. Through ROC analysis, we found AUC of 0.791 for HIP1R differentiating GC patients from healthy controls. Moreover, in subgroup analysis, the AUC of HIP1R for lymph node metastasis and advanced GC stages reached 0.801 and 0.806. These results indicate that HIP1R may be a potential diagnostic marker for GC.

Acquired apoptosis resistance is a hallmark of most cancers.^40^ For this reason, the discovery of potential biomarkers or drugs to target this process holds great interest. Our study implicates that HIP1R acts as a tumor suppressor gene promoting GC cell apoptosis. This biological function is in agreement with previous studies. It was reported that HIP1R interacts with BCL2L10 and activates caspase**‐**9, with HIP1R overexpression increasing the correlation of BCL2L10 with caspase**‐**9 and disrupting mitochondrial membrane potential.^41^ Consistent with this, Wang et al^23^ found that disruption of HIP1R could inhibit tumor cell sensitivity to T cell killing and lead to decreased tumor cell apoptosis. As we know, the structure of HIP1R protein is composed of 3 parts, an N‐terminally localized ANTH domain, a central coiled‐coil domain, and a talin‐like domain at the C‐terminus. Previous studies found that the ANTH domain of HIP1R could bind PtdIns‐3,4‐P_2_.^16^ PtdIns‐3,4‐P_2_ has been confirmed to induce the dimerization then enhance the phosphorylation of Akt.^42^, ^43^ Because both HIP1R and Akt could bind PtdIns‐3,4‐P_2_, we hypothesize that overexpression of HIP1R could competitively bind PtdIns‐3,4‐P_2_ to prevent phosphorylation of Akt. We further observed that HIP1R overexpression markedly reduced p**‐**Akt and p‐mTOR levels in GC cells compared with controls, while knockdown HIP1R expression correlated with increased expression of p‐Akt and p‐mTOR in our studies. The PI3K/Akt signaling pathway plays important roles in tumor cell survival and apoptosis. For instance, inactivation of the PI3K/Akt pathway can induce apoptosis of breast carcinoma cells.^44^ Thus, we speculate that HIP1R can activate the cell death pathway. In our studies, we found that overexpression of HIP1R led to increased expression of Cleaved Caspase‐9 and Bak, while silencing HIP1R expression can also downregulated the expression of Cleaved Caspase‐9 and Bak. Our results were coincidence with previous studies.^41^ Moreover, we performed CCK8 assays, colony formation assays, and EdU labeling assays to examine the regulation of HIP1R on the proliferation of GC cells. The proliferation assays all showed that GC proliferation rate was inhibited by overexpression HIP1R and increased by knockdown HIP1R. These results suggested that HIP1R could promote apoptosis of GC cells, subsequently blocking tumor growth.

Our migration and invasion assays indicated that HIP1R overexpression could inhibit HGC27 cell motility compared with negative control, while siRNA depletion HIP1R could promote MGC803 cell motility compared with control siRNA. This biological effect coincided with our clinical analysis results. Besides, we also found that the expression of HIP1R suppresses the expression of Vimentin and enhanced the expression of E‐cadherin. Based on our previous results, we speculated that HIP1R may suppress EMT via the AKT pathway and suppresses cell invasion. However, Rice et al^26^ reported that the microRNA**‐**23b/**‐**27b cluster indirectly regulated HIP1R, while ectopic expression of HIP1R increased migration and invasion in human prostate cancer cells. These findings indicate that HIP1R acts as both a tumor promoter and a suppressor gene, highlighting the need to define specific gene roles in certain cancer types. It is still important to thoroughly understand the molecular mechanisms by which HIP1R regulates its differential biological effects in GC and other cancer types. It was reported that activating the PI3K/Akt signaling pathway promotes migration and invasion of pancreatic ductal adenocarcinoma,^45^ similar to our results. Therefore, we speculate that HIP1R expression has properties to inhibit cancer cell migration and invasion.

## 5 CONCLUSION

In conclusion, our studies indicate that HIP1R is downregulated in human GC in association with N stage. We also demonstrated the diagnostic efficiency of HIP1R in GC. Furthermore, the expression of HIP1R could promote GC cell apoptosis and inhibit proliferation, migration and invasion, possibly through modulating Akt. We believe that HIP1R downregulation may identify high**‐**risk patients and serve as a new therapeutic target for GC.
